# Potential Cardiovascular Risks of Phenethylamine Analogues in Food Supplements: Evidence from Vasocontraction of Rat Artery Segments

**DOI:** 10.1007/s12012-026-10109-8

**Published:** 2026-04-04

**Authors:** Nicole E. T. Pinckaers, W. Matthijs Blankesteijn, Peter Leenders, Ellen Weltjens, Antoon Opperhuizen, Frederik-Jan van Schooten, Misha Vrolijk

**Affiliations:** 1 Department of Pharmacology and Toxicology, Maastricht University, Universiteitssingel 50, 6229 ER Maastricht, The Netherlands; 2 Research Institute of Nutrition and Translational Research in Metabolism (NUTRIM), Maastricht University, Maastricht, The Netherlands; 3 Cardiovascular Research Institute Maastricht (CARIM), Maastricht University, Maastricht, The Netherlands; 4 Office for Risk Assessment and Research, Netherlands Food and Consumer Product Safety Authority (NVWA), Utrecht, The Netherlands

**Keywords:** Phenethylamines, Food supplements, Vasocontractility, Mesenteric artery, Aorta, Renal artery

## Abstract

Phenethylamine (PEA) and alkylamine (AA) analogues are frequently present in pre-workout food supplements and share structural similarity with amphetamine and the endogenous catecholamines (nor)adrenaline and dopamine, suggesting potential sympathomimetic activity. The present study investigated the vasoactive properties of PEA and AA analogues in isolated Wistar Kyoto rat mesenteric arteries, renal arteries and aorta. Our findings provide evidence that several analogues induced concentration-dependent contraction of isolated blood vessels, with potency (EC_50_) values ranging from 0.906 µM to 550 µM. *p*-Synephrine and *p*-octopamine were the only two substances that induced contraction of the mesenteric artery segments, whereas 6 out of 12 PEA analogues, including *p*-synephrine, *p*-octopamine, halostachine, β-methylphenethylamine, phenethylamine and dimethylphenethylamine, induced contraction of aorta and renal artery segments. These effects were largely attenuated by the α_1_-adrenergic receptor (ADR) antagonist prazosin, implicating ADRα_1_ involvement, while partial modulation by the trace amine associated receptor 1. The demonstrated vasocontractility of these compounds highlights the potential influence on the vascular tone and blood pressure regulation and the deleterious consequences for cardiovascular health in humans. These risks may be particularly pronounced in exercising individuals that have an already activated sympathetic nervous system or those with underlying cardiovascular disease.

## Introduction

**Fig. 1:**
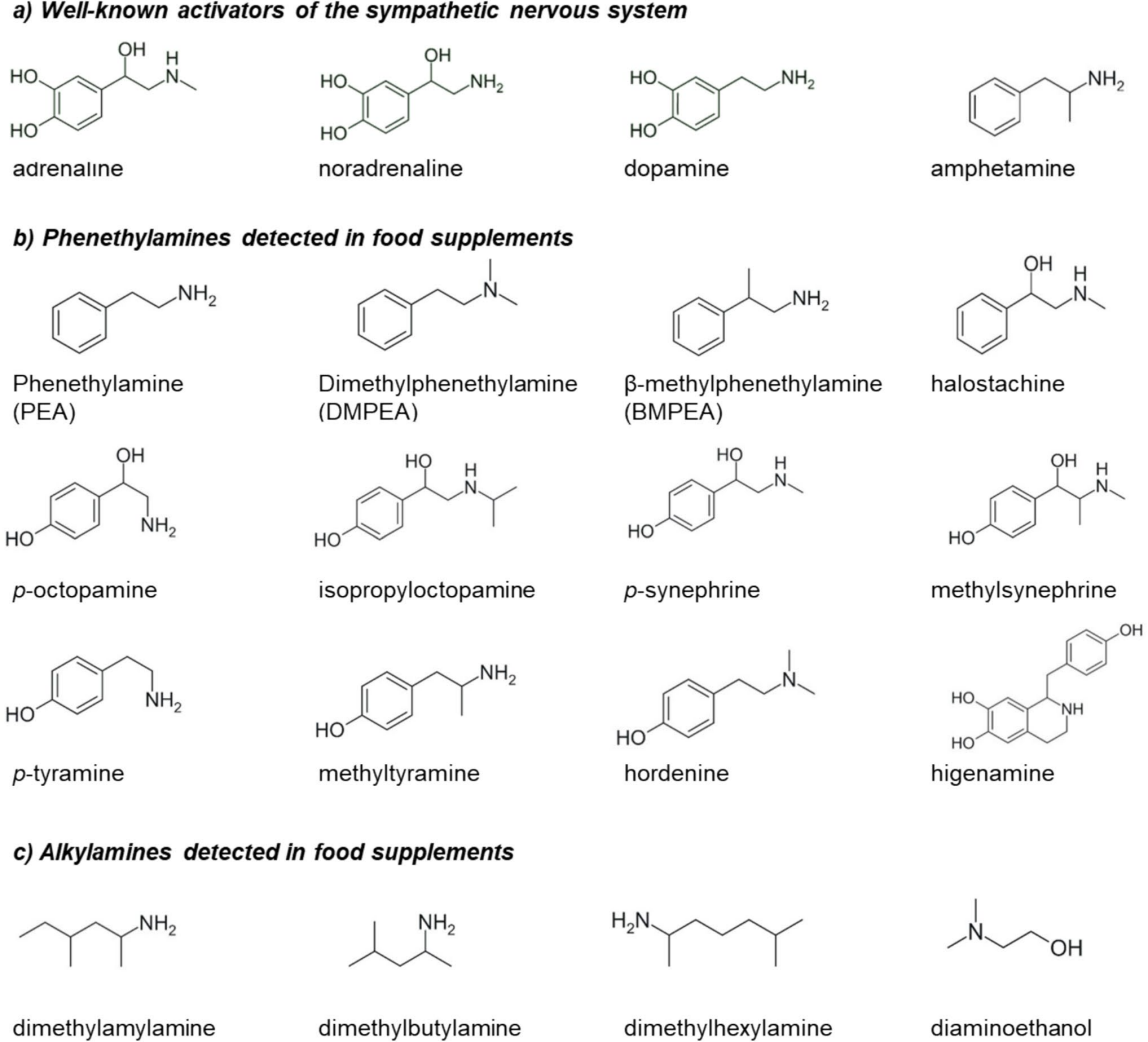
Molecular structures of **a** well-known activators of the sympathetic nervous system, **b** phenethylamine (PEA) and its analogues and **c** alkylamines (AAs) detected in food supplements

Food supplements marketed for sport performance enhancement and weight loss are becoming increasingly popular worldwide [1, 2]. These supplements are often perceived by consumers as “natural” and therefore safe, yet many products contain pharmacologically active compounds. A prominent group of substances present in such supplements are phenethylamine (PEA) analogues and alkylamine (AA) analogues, including synephrine, dimethylamylamine and dimethylbutylamine [3, 4]. Structurally, these substances are closely related to the synthetic stimulant amphetamine and to the endogenous catecholamines (nor)adrenaline and dopamine (Fig. 1). This structural similarity suggests potential sympathomimetic activity, raising concerns about their impact on the cardiovascular system.

Despite their widespread use and presence in food supplements, the safety of PEA and AA analogues is scarcely studied, and their use remains largely unregulated [3]. Alarmingly, multiple adverse cardiovascular events, such as hemorrhagic stroke, tachycardia and myocardial infarction, have been linked to the use of such supplements [5–8]. This urges the need for more accurate hazard characterization of these substances to improve risk assessment and regulatory decision-making, and ultimately to protect consumer health.

The reported cardiovascular toxicity is suggested to be caused by the ability of the PEA and AA analogues to mimic sympathetic nervous system activation via multiple mechanisms. We have previously shown that PEA and several of its analogues are capable of activating human adrenergic receptors (ADRs) and the trace amine associated receptor 1 (TAAR1) in vitro [9]. ADRα_1_ is mainly expressed in the vasculature and plays a key role in blood pressure regulation by inducing vascular smooth muscle contraction [10]. The more recently discovered TAAR1 has also been identified in peripheral tissues (e.g. aorta and kidney), where it is hypothesized to contribute directly and indirectly to vascular smooth muscle cell contraction [11–13]. Vascular smooth muscle cell contraction reduces the blood vessel diameter, thereby increasing vascular resistance and blood pressure, and ultimately influencing cardiac output [14].

Although activation of ADRs and TAAR1 by several PEA analogues has been demonstrated in vitro, the direct functional consequences of this receptor activation for vascular tone remain largely unknown. In particular, little is known whether these compounds can induce vasoconstriction in intact vascular tissue and whether such effects differ between vascular beds with distinct physiological roles in the circulation.

Therefore, the present study systematically investigated the vasoactive properties of PEA and AA analogues (Fig. 1b and c) in three functionally distinct rat arteries: mesenteric arteries, aorta and renal arteries. By combining concentration–response analysis with mechanistic investigations of isolated artery segment contraction force, assessed in the presence of the ADRα_1_ antagonist prazosin and TAAR1 antagonist N-(3-ethoxyphenyl)−4-pyrrolidin-1-yl-3-(trifluoromethyl)benzamide (EPPTB), this study links molecular receptor activation to physiologically relevant vascular responses. This approach provides new insights into the cardiovascular risks posed by PEA and AA analogues commonly found in food supplements.

## Materials and Methods

### Animals, Ethical Statement and Husbandry

Female and male Wistar Kyoto (WKY) rats (Charles River Laboratories) with an average age of 23.8 weeks (SD = 7.2 weeks) were socially housed in cages in a controlled room (temperature = 20.8 ± 0.312 °C; humidity = 55.0 ± 5.81%) with a reverse light–dark cycle with free access to food and drinking water. Lights were on from 07:00 p.m. to 07:00 a.m. All procedures with animals were carried out with the approval of the Dutch National Committee for Animal Experimentation (Centrale Commissie Dierproeven, CCD) and the Animal Welfare Body of Maastricht University (License number: AVD10700202216336). A total of 11 male and 9 female rats was used in this study.

### Chemicals

Phenylephrine hydrochloride (≥ 99%), acetylcholine hydrochloride (≥ 99%), β-methylphenethylamine (BMPEA) (99%), phenethylamine (99.7%), tyramine hydrochloride (≥ 98%), halostachine (99%), hordenine (≥ 97.5%), octopamine (≥ 95%), dimethylbutylamine (98%), dimethylhexylamine (99%), dimethylaminoethanol (99.8%), magnesium sulfate (≥ 99.5%), calcium chloride (≥ 93.5%), D-(+)-glucose, N-(3-ethoxyphenyl)−4-pyrrolidin-1-yl-3-(trifluoromethyl)benzamide (EPPTB; > 95%) and HEPES were purchased from Sigma-Aldrich (St. Louis, USA). Synephrine hydrochloride (99.6%) and higenamine hydrochloride (98.98%) were purchased from MedChemExpress (Monmouth Junction, USA). Methyltyramine (97%) and 1,3-dimethylamylamine hydrochloride (95%) were purchased from Apollo Scientific (Stockport, United Kingdom). Methylsynephrine hydrochloride (99.8%) was purchased from Mikromol (Luckenwalde, Germany), isopropyloctopamine hydrochloride (98%) from Toronto Research Chemicals (Toronto, Canada), N,N-dimethylphenethylamine (DMPEA) hydrochloride (> 99%) from GLPBio (Montclair, USA) and dimethylsulfoxide (DMSO) from Carl Roth (Karlsruhe, Germany). Sodium chloride, monopotassium phosphate (≥ 99.5%) and sodium bicarbonate (≥ 99%) were purchased from Merck (Darmstadt, Germany). Potassium chloride was purchased from Boom B.V. (Meppel, The Netherlands). Prazosin hydrochloride was purchased from EDQM (Strasbourg, France). Methyltyramine and higenamine hydrochloride were dissolved in DMSO. All the other chemicals were dissolved in Krebs Ringer bicarbonate buffer (KRB) (composition in mM: NaCl 118.5, KCl 4.7, MgSO_4_ 1.2, KH_2_PO_4_ 1.2, NaHCO_3_ 25, CaCl_2_ 2.5 and glucose 5.5).

### Artery Isolation and Mounting into a Small Vessel Wire Myograph

Rats were euthanized by a pentobarbital injection (200 mg/kg body weight). The mesentery, renal artery and thoracal aorta were dissected and immediately transferred to a cold HEPES solution (composition in mM: NaCl 143.3, KCl 4.7, MgSO_4_ 1.2, KH_2_PO_4_ 1.2, CaCl_2_ 2.5, glucose 5.5 and HEPES 15.). Second-order branches of the mesenteric artery were isolated. All arteries were cut into segments of 0.9 to 2 mm and mounted in a small vessel wire myograph (Danish Myograph Technology, Aarhus, Denmark). Two wires, with a diameter of 40 µm, were passed through the vessel lumen. One wire was attached to a force transducer to measure wall tension changes, and the other wire was connected to a micropositioner to control the distance between the two wires. The organ baths of the myograph were filled with 7 mL KRB and arteries were maintained at 37° C and continuously oxygenated with 5% CO_2_.

Vessels were passively stretched to the diameter corresponding to a transmural pressure of 13.3 kPa, as defined by the DMT normalization procedure (Vol. 2.1). Contractile responses were expressed as the increase in wall tension (in mN/mm), by dividing force (mN) by two times the artery segment length (mm), relative to the maximal contractile responses induced by phenylephrine.

### Contractility of Isolated Artery Segments

After the vessel normalization procedure, the artery segments were washed with KRB and equilibrated for at least 30 min. Then, the contractility of the artery segments were studied by adding cumulative concentrations of phenylephrine (0.3, 1, 3, 10, 30 and 100 µM). The endothelial function of mesenteric arteries was tested by adding 1 µM acetylcholine once maximal contractility was reached. This induced an endothelium-dependent relaxation of the mesenteric arteries with an intact endothelium, which was an inclusion criterium for this study. Subsequently, the artery segments were washed three times with KRB and compound exposure was started when the wall tension was back to baseline condition. Contractility of the artery segments by the PEA and AA analogues was studied in a cumulative manner with concentrations ranging from 0.03 to 1000 µM. In addition, ADRα_1_-mediated contractility by PEA and AA analogues was studied with a 15-min pre-incubation with 1 µM of the ADRα_1_-antagonist prazosin. TAAR1-mediated contractility by PEAs was studied with a 15-min pre-incubation with 30 µM of the TAAR1-antagonist EPPTB.

### Data Analysis

Vasocontractile responses were acquired with LabChartPro software (AD Instruments, v8.1.30). Data are shown as mean ± SD. Vasocontractile responses are presented as percentage of the maximal response to phenylephrine in each artery segment. Concentration–response curves were fitted to non-linear regression curves with a standard Hill slope of 1 (GraphPad Software, version 10.1.2, San Diego, USA). The EC_50_ and E_max_ values are presented as an average of the independent experiments with 95% confidence intervals (CI).

## Results

### Contractility of Isolated Mesenteric Artery Segments

**Fig. 2:**
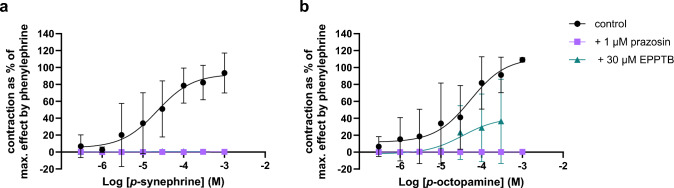
Contractility of mesenteric artery segments by **a** *p*-synephrine (n = 4*) and **b** *p*-octopamine (n = 4*) without (control; circles) and with α_1_ adrenergic-receptor antagonist prazosin (1 µM; n = 2; squares) or TAAR1-antagonist N-(3-ethoxyphenyl)−4-pyrrolidin-1-yl-3-(trifluoromethyl)benzamide (EPPTB; 30 µM; n = 2; triangles); data are shown as mean ± SD; vasocontractile responses are presented as percentage of the maximal response to phenylephrine in each artery. * treatments with *p*-synephrine and *p*-octopamine were performed in mesenteric artery segments from 6 different animals, of which in 2 there was no response to these compounds (data not shown)

Phenylephrine was used as a reference compound and induced a maximal contraction of the isolated mesenteric artery segments (E_max_ = 100%) in a concentration-dependent manner. Contraction of mesenteric artery segments was only induced by *p*-synephrine (E_max_ = 92.3%) and *p*-octopamine (E_max_ = 112%), with EC_50_ values of 22.4 µM and 53.2 µM, respectively (Fig. 2; Table 1). These effects were observed in artery segments from 4 out of the 6 animals (in 2 artery segments there was no response to these compounds, data not shown). The effects of *p*-synephrine and *p*-octopamine were completely blocked by prazosin. EPPTB antagonized the effect of *p*-synephrine completely, while the effect of *p*-octopamine was only partially blocked. None of the other selected PEA and AA analogues induced vasocontraction of the mesenteric arteries at concentrations up to 300 µM (Fig. 5, Appendix).

**Table 1:** Contractile efficacy (E_max_) and potency (EC_50_) values of *p*-synephrine, *p*-octopamine, halostachine, β-methylphenethylamine (BMPEA), phenethylamine (PEA) and dimethylphenethylamine (DMPEA) on isolated rat mesenteric artery segments, aorta segments and rat renal artery segments without and with prazosin or EPPTB; the efficacy is expressed as % of the maximal response induced by phenylephrine (E_max_ = 100%); values are shown as an average (95% confidence interval) of 2–4 independent experiments; an asterisk (*) indicates that it was not possible to establish E_max_ and EC_50_ values; n.d. indicates no data is available; a question mark (?) indicates it was not possible to establish an upper and/or lower value of the 95% confidence interval

	Mesenteric artery		Aorta		Renal artery	
	E_max_ (%)	EC_50_ (µM)	E_max_ (%)	EC_50_ (µM)	E_max_ (%)	EC_50_ (µM)
***p*-synephrine**	92.3 (74.1–115)	22.4 (6.00–82.8)	86.9 (78.4–95.6)	0.906 (0.478–1.74)	97.9 (88.7–108)	2.29 (0.893–5.86)
+ prazosin	*	*	*	*	22.2 (9.23-?)	846 (130-?)
+ EPPTB	*	*	79.1 (68.8–90.9)	7.19 (2.44–21.8)	73.9 (57.2–95.6)	11.1 (1.24–77.7)
***p*-octopamine**	112 (86.5–147)	53.2 (13.6–210)	88.7 (81.2–96.6)	2.05 (0.972–4.28)	90.2 (80.5–101)	6.06 (2.24–16.0)
+ prazosin	*	*	*	*	*	*
+ EPPTB	*	*	152 (132–174)	6.77 (2.48–18.6)	71.0 (58.8–87.3)	5.35 (0.499–56.5)
**halostachine**	*	*	99.9 (86.9–114)	1.29 (0.433–4.16)	61.8 (51.0–74.0)	1.37 (0.204–9.11)
+ prazosin	n.d	n.d	*	*	*	*
+ EPPTB	n.d	n.d	66.4 (40.1–120)	19.2 (0.708–453)	56.6 (45.1–71.1)	9.42 (1.50–60.3)
**BMPEA**	*	*	109 (68.3–589)	550 (171–7199)	64.9 (48.6–90.2)	16.9 (1.77–172)
+ prazosin	n.d	n.d	36.2 (11.0-?)	*	70.0 (51.9–119)	31.6 (1.97–464)
+ EPPTB	n.d	n.d	*	*	20.9 (0.359-?)	10.7 (?-?)
**PEA**	*	*	98.1 (90.9–106)	5.57 (3.45–9.01)	85.8 (78.6–93.7)	3.80 (1.45–9.67)
+ prazosin	n.d	n.d	20.6 (6.22-?)	51.5 (?)	99.5 (72.8–138)	69.5 (19.6–257)
+ EPPTB	n.d	n.d	90.1 (50.7–486)	116 (6.06–49370)	63.9 (50.4–80.2)	3.11 (?−68.7)
**DMPEA**	*	*	86.5 (69.0–110)	148 (71.8–317)	50.9 (31.5-?)	47.7 (0.442-?)
+ prazosin	n.d	n.d	*	*	n.d	n.d
+ EPPTB	n.d	n.d	199 (88.00-?)	1330 (233-?)	n.d	n.d

### Contractility of Isolated Aorta Segments

**Fig. 3:**
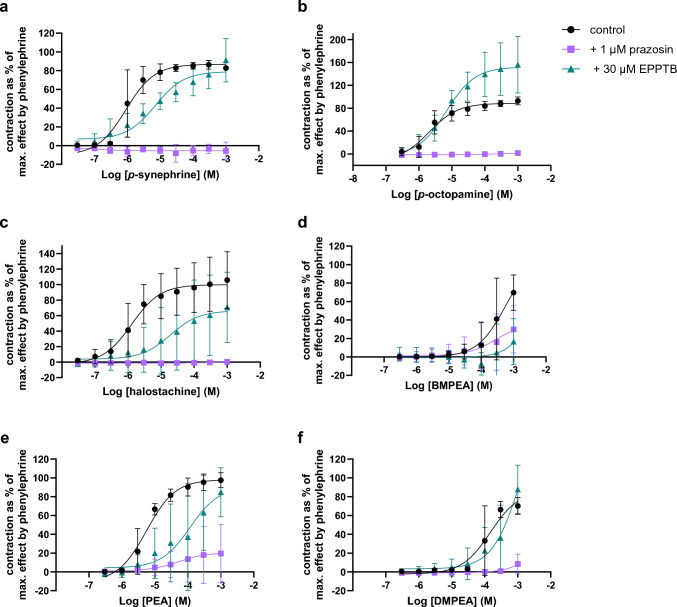
Concentration-response relationships (mean ± SD) of compounds* that induced contraction of isolated aorta segments of WKY rats without (control; circles) and with α1 adrenergic-receptor antagonist prazosin (1 µM; squares) or TAAR1-antagonist EPPTB (30 µM; triangles); data are presented as percentages of the maximal effect obtained by phenylephrine * **a** *p*-synephrine (n = 3), +prazosin (n = 3), +EPPTB (n = 3); **b** p-octopamine (n = 3), +prazosin (n = 3), + EPPTB (n = 3); **c** halostachine (n = 4), +prazosin (n = 3), + EPPTB (n=3); **d** β-methylphenethylamine (BMPEA; n = 6), + prazosin (n = 3), +EPPTB (n = 4); **e** phenethylamine (PEA; n = 3) +prazosin (n = 3), +EPPTB (n = 3); **f** dimethylphenethylamine (DMPEA; n = 4) +prazosin (n = 3), +EPPTB (n = 3)

Contractility of isolated aorta segments was induced in a concentration-dependent manner by *p*-synephrine (EC_50_: 0.906 µM; E_max:_ 86.9%), *p*-octopamine (EC_50_: 2.05 µM; E_max:_ 88.7%), halostachine (EC_50_: 1.29 µM; E_max:_ 99.9%), BMPEA (EC_50_: 550 µM; E_max:_ 109%), PEA (EC_50_: 5.57 µM; E_max:_ 98.1%) and DMPEA (EC_50_: 148 µM; E_max:_ 86.5%;Fig. 3a-f, Table 1). Prazosin completely inhibited the effects of *p*-synephrine, *p*-octopamine and halostachine (Fig. 3a-c) and partially inhibited the effects of BMPEA, PEA and DMPEA (Fig. 3d-f). EPPTB led to a rightward displacement of the concentration–response curves of *p*-synephrine, halostachine, BMPEA, PEA and DMPEA. Interestingly, the effects of *p*-octopamine seemed to be enhanced by EPPTB. *p*-Tyramine only induced vasocontraction at higher concentrations (100–1000 µM; Fig. 6a, Appendix). Additionally, the 4 AA analogues inconsistently induced contractility, with a high variation between the independent experiments (Fig. 6g-j, Appendix). The other PEA and AA analogues did not induce contractility up to concentrations of 1 mM (Fig. 6, Appendix).

### Contractility of Renal Artery Segments

**Fig. 4:**
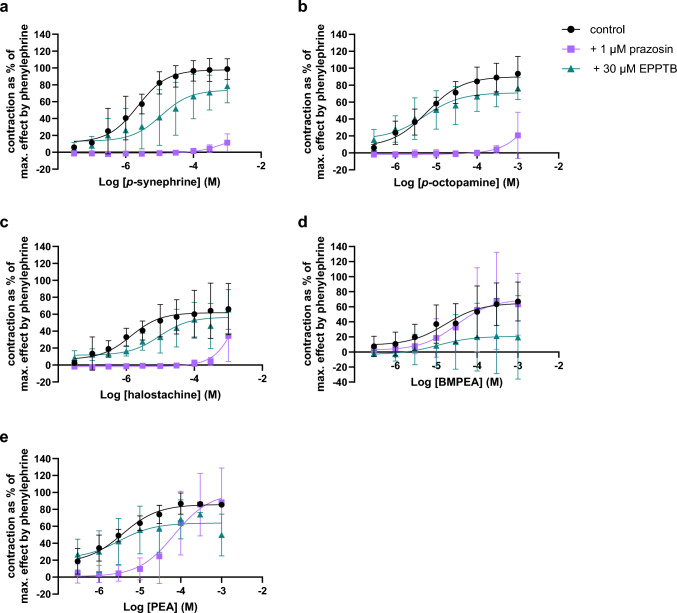
Concentration–response relationships (mean ± SD) of compounds* that induced contraction of isolated renal artery segments of WKY rats without (control; circles) and with α1 adrenergic-receptor antagonist prazosin (1 µM; squares) or TAAR1-antagonist N-(3-ethoxyphenyl)-4-pyrrolidin-1-yl-3-(trifluoromethyl)benzamide (EPPTB; 30 µM; triangles); data are presented as percentages of the maximal effect obtained by phenylephrine. * **a ***p*-synephrine (n = 3), +prazosin (n = 3), +EPPTB (n = 3); **b ***p*-octopamine (n = 3), +prazosin (n = 3), +EPPTB (n = 3); **c** halostachine (n = 3), +prazosin (n = 3), + EPPTB (n = 3); **d **β-methylphenethylamine (BMPEA; n = 4), +prazosin (n = 3),  +EPPTB (n = 3); **e** phenethylamine (PEA; n = 3) +prazosin (n = 3), +EPPTB (n = 3)

**Fig. 5:**
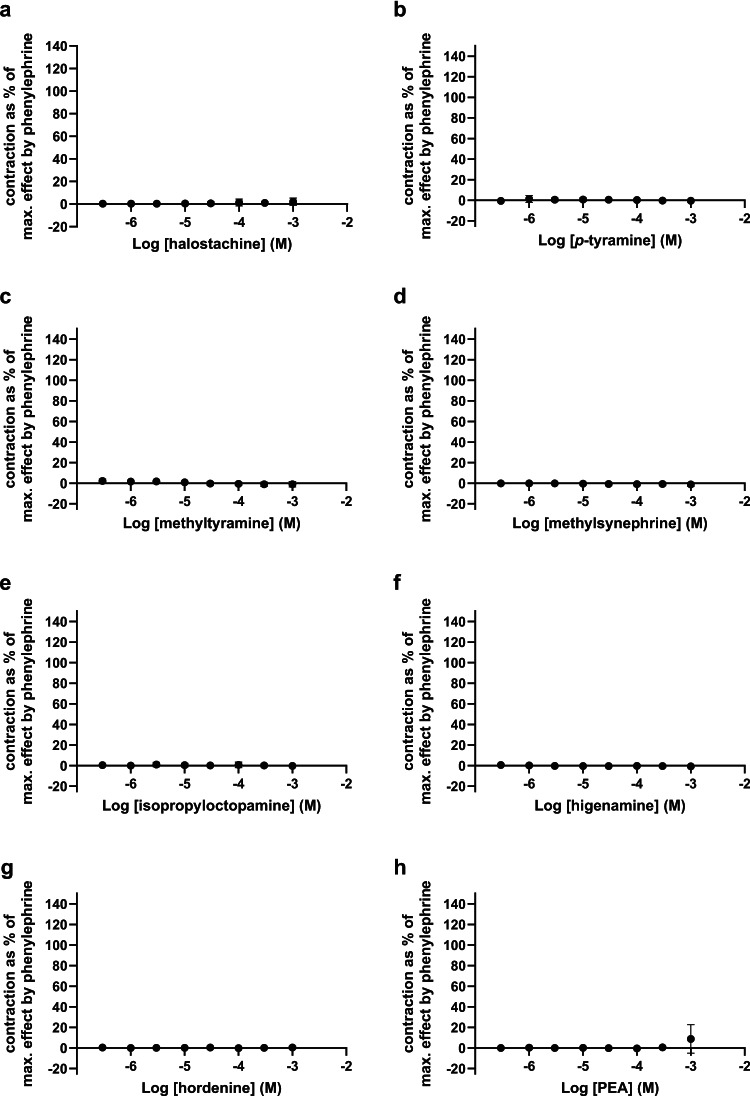

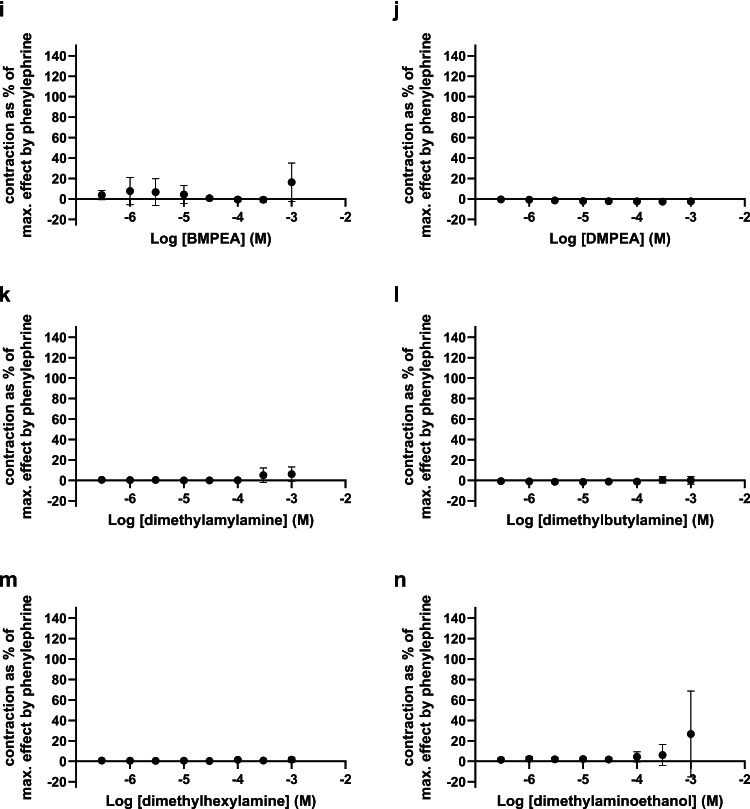
Vasocontractile response of mesenteric arteries in the presence of phenethylamine and alkylamine analogues **a** halostachine, **b** *p*-tyramine, **c** methyltyramine, **d** methylsynephrine, **e** isopropyloctopamine, **f** higenamine, **g** hordenine, **h** phenethylamine (PEA), **i** β-methylphenethylamine (BMPEA), **j** dimethylphenethylamine (DMPEA), **k** dimethylamylamine, **l** dimethylbutylamine, **m** dimethylhexylamine, **n** dimethylaminoethanol (all n = 4); data are shown as mean ± SD; vasocontractile responses are presented as percentage of the maximal response to phenylephrine in each artery

**Fig. 6:**
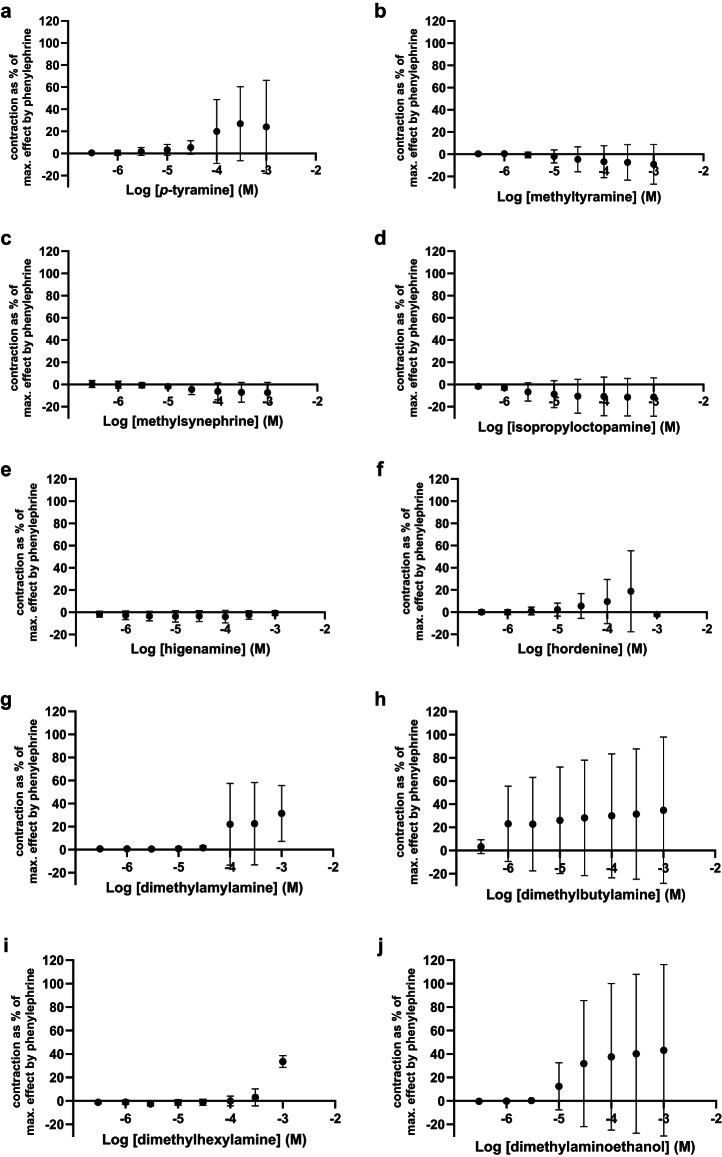
Vasocontractile response of rat aorta segments in the presence of the phenethylamine and alkylamine analogues **a** *p*-tyramine, **b** methyltyramine, **c** methylsynephrine, **d** isopropyloctopamine, **e** higenamine, **f** hordenine, **g** dimethylamylamine, **h** dimethylbutylamine, **i** dimethylhexylamine, **j** dimethylaminoethanol; data are shown as mean ± SD (all n = 3); vasocontractile responses are presented as percentage of the maximal response to phenylephrine in each artery

**Fig. 7:**
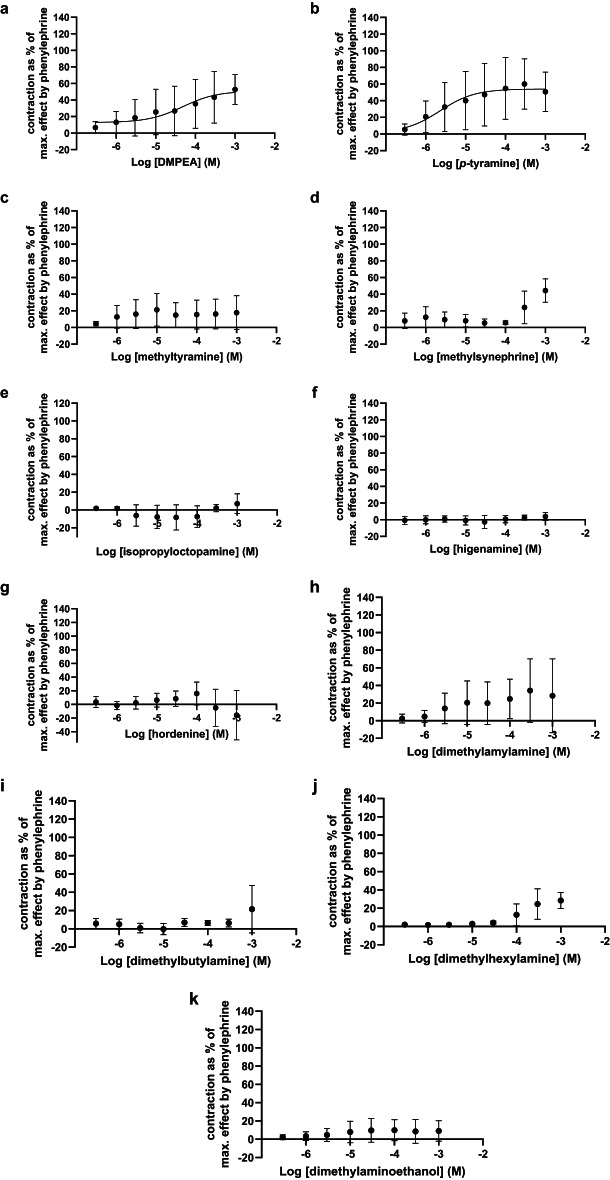
Vasocontractile response of rat renal arteries in the presence of the phenethylamine and alkylamine analogues **a** dimethylphenethylamine (DMPEA; n = 4), **b** *p*-tyramine (n = 5), **c** methyltyramine (n = 3), **d** methylsynephrine (n = 3), **e** isopropyloctopamine (n = 3), **f** higenamine (n = 3), **g** hordenine (n = 3), **h** dimethylamylamine (n = 3), **i** dimethylbutylamine (n = 3), **j** dimethylhexylamine (n = 3), **k** dimethylaminoethanol (n = 3); data are shown as mean ± SD; vasocontractile responses are presented as percentage of the maximal response to phenylephrine in each artery

Contractility of isolated renal arteries was induced in a concentration-dependent manner by *p*-synephrine (EC_50_: 2.29 µM; E_max_ = 97.7%), *p*-octopamine (EC_50_: 6.06 µM; E_max_ = 90.2%), halostachine (EC_50_: 1.37 µM; E_max_ = 61.8%), BMPEA (EC_50_: 16.9 µM; E_max_ = 64.9%) and PEA (EC_50_: 3.80 µM; E_max_ = 85.8%; Fig. 4a-e and Table 1). In addition, DMPEA (E_max_ = 50.9%) and *p*-tyramine (E_max_ = 54.1%) induced contraction but with a smaller and more varying effect size (Fig. 7a-b, Appendix). Prazosin almost completely inhibited contraction by *p*-synephrine, *p*-octopamine and halostachine. However, at a concentration of 1000 µM, *p*-synephrine, *p*-octopamine and halostachine induced minor contractile effects in the presence of prazosin (E_max_ = 22.2%, 20.7% and 34.5%, respectively). Prazosin did not affect the contraction induced by BMPEA (E_max_ = 70.0%) and PEA (E_max_ = 99.5%), but it reduced PEA’s potency (EC_50_ = 69.5 µM; Fig. 4e). EPPTB slightly inhibited the contractile effects of *p*-synephrine (E_max_ = 73.9%), *p*-octopamine (E_max_ = 71.0%), halostachine (E_max_ = 56.6%), BMPEA (E_max_ = 20.9%), and PEA (E_max_ = 63.9%) and reduced the potency of *p*-synephrine (EC_50_ = 11.1 µM) and halostachine (EC_50_ = 9.42 µM; Table 1, Fig. 4a-e).

## Discussion

The aim of the present study was to investigate the contractile properties of PEA and AA analogues on isolated rat mesenteric artery, renal artery and aorta segments. To our knowledge, this study is the first systematic comparison of the vasocontractile effects of a broad panel of PEA and AA analogues across multiple arterial beds. These compounds, which are structurally related to endogenous catecholamines and synthetic sympathomimetics and often found in sports supplements, have previously been shown to activate human ADRs and TAAR1 in vitro, implying that they could be vasoactive and thereby potentially affecting cardiovascular health in vivo [9]. While previous studies mainly focused on receptor activation in different systems or isolated examples of individual compounds, the present work demonstrates that these substances can directly induce functionally relevant vasocontraction in intact vascular tissue. Importantly, the magnitude and potency of these responses differed substantially between resistance arteries and larger conduit arteries, highlighting the vascular bed-specific nature of their pharmacological effects**.** Our findings support the potential of the analogues to modulate cardiovascular function in vivo. Most of the observed effects were mediated by ADRα_1_, as demonstrated by prazosin antagonism, although TAAR1 also appeared to contribute. The systematic evaluation of multiple structurally related compounds across three vascular beds provides a functional framework for prioritizing substances that may pose the greatest cardiovascular risk.

Here we studied the effects of PEA and AA analogues on three vascular beds with distinct physiological roles. Vasomodulation of these vessels plays a crucial role in cardiovascular health by regulating blood pressure, oxygen delivery, and nutrient transport throughout the body. Mesenteric arteries, classified as resistance arteries, are the smallest and are considered muscular arteries, that play a major role in regulating systemic vascular resistance and blood pressure. They are densely innervated with sympathetic nerves, enabling indirect sympathetic activation by the release of noradrenaline from nerve endings [15–17]. Moreover, their vascular smooth muscle cells are known to be extensively expressing ADR, which stimulates contraction of these cells upon activation by noradrenaline or other ADRα_1_ agonists [18–20]. This explains the vasocontractile responses of the selective ADRα_1_ agonist phenylephrine in the current study. Surprisingly, only two of the studied PEA analogues (i.e. *p*-synephrine and *p*-octopamine) induced contraction of the mesenteric artery segments, while other analogues were inactive at comparable concentrations. These findings are consistent with previous studies demonstrating vasodilatory responses of some of these PEA analogues (*p*-tyramine, *p*-octopamine and PEA) in mesenteric arteries pre-contracted with phenylephrine [21, 22], which were inhibited by the non-selective nitric oxide synthase (NOS) inhibitor L-NAME [21]. This suggests that the vasodilatation caused by PEA and the two analogues is likely mediated via NO-release, which could abolish potential vasocontractile actions by these compounds. As the present study focused on the vasocontractile responses of isolated arteries by these compounds, future studies specifically designed to investigate endothelial signaling pathways may provide additional mechanistic insight. The discrepancy with our results, where *p*-octopamine and *p*-synephrine induced vasocontraction in a subset of experiments, might be explained by regional heterogeneity in endothelial NOS expression or NO availability within the mesenteric arterial bed and even within mesenteric arteries from the same branch [23–26].

Given the regional variability in vasocontractile responses, we also included two larger arterial beds in the current study: the renal artery and the aorta. In contrast to the mesenteric arteries, the renal artery and aorta exhibited robust vasocontraction in response to a broader range of PEA analogues, including halostachine, BMPEA, PEA, DMPEA, *p*-tyramine, *p*-synephrine and *p*-octopamine, of which the latter two were more potent in these larger arteries compared to the mesenteric arteries. Similar results have previously been reported for noradrenaline, which showed higher potency in the aorta than in mesenteric arteries isolated from rat and rabbit [27, 28]. The effects caused by *p*-synephrine and *p*-octopamine were ADRα_1_-mediated in all three vessel types, which is in line with previous studies performed with porcine renal arteries and rat mesenteric arteries [29–31]. We demonstrated that PEA and multiple analogues induce vasocontraction of renal artery segments, which implies potential blood pressure upregulation and harmful effects in vivo by these compounds. Our findings align with a previous study that demonstrated dose-dependent blood pressure elevations in rats after subcutaneous BMPEA administration, which were also antagonized by prazosin [32]. The renal artery is a muscular artery that is essential in systemic blood pressure regulation by controlling renal blood flow and activating the renin–angiotensin–aldosterone system (RAAS) [33, 34]. Severe or prolonged vasocontraction and -constriction of renal arteries could compromise renal perfusion and ultimately lead to renal dysfunction or failure. Indeed, the use of synephrine has been associated with cases of renal artery occlusion and renal failure [6, 35]. Such risks may be higher in individuals with pre-existing cardiovascular or renal pathologies or during exercise when sympathetic tone is already increased. This is for example the case for heart failure patients, that have an already increased renal vasoconstriction [36] and for patients with peripheral arterial disease, that showed increased renal vasoconstriction during exercise [37].

The aorta is the largest artery, containing an elastic wall, which enables its buffering capacity by expanding during systolic heart contractions and recoiling during diastolic contractions [38]. Traditionally it was considered solely as a conduit artery. However, accumulating evidence indicates that it also plays an active role in hemodynamic regulation [39]. Constriction of the aorta could increase the pulse pressure and systolic blood pressure. Our data showed that PEA, 7 of its analogues and the AA analogues induce vasocontraction of isolated aorta segments, which were mainly ADRα_1_-mediated. This implies a potential rise in pulse pressure and systolic blood pressure when exposed to these compounds. Since the role of the aorta in cardiovascular homeostasis is crucial, changes in its functioning and structure could be life-threatening. High aortic blood pressure or an increased shear stress could result in damage to its wall and in the end to aortic dissection [40–42]. In fact, an aortic dissection has been observed in a 38-year-old patient after chronically using a workout supplement containing synephrine [43].

The receptor mechanisms underlying the vasoactive effects of the PEA analogues appear heterogenous. While prazosin consistently attenuated vasocontraction across the different vessel types and the different analogues, the TAAR1 antagonist EPPTB gave variable results. In mesenteric arteries, EPPTB completely inhibited the effects of *p*-synephrine but only partially inhibited vasocontraction by *p*-octopamine. However, these results should be interpreted with caution, as the sample size of the studies with the different antagonists in mesenteric arteries was smaller (n = 2). In the renal artery and aorta, EPPTB reduced the potency rather than the maximal effect of several analogues (e.g. *p*-synephrine), whereas there was no inhibitory effect on the vasocontractile effects of *p*-octopamine. Interestingly, EPPTB even seems to enhance the vasocontractile effects of *p*-octopamine in the aorta, possibly reflecting receptor crosstalk or compensatory signaling. Additionally, the potency of the known TAAR1-agonist PEA was reduced by EPPTB in the aorta, whereas there was hardly any impact on the renal artery, supporting the higher TAAR1 expression in the aorta which has been demonstrated previously [11, 13]. In addition, PEA’s response was largely reduced by prazosin in the aorta (E_max_ = 20.6%), whereas its efficacy was not affected by prazosin in the renal artery (E_max_ = 99.5%), suggesting that vasocontraction of the various vascular beds occurs via various mechanisms. Our findings highlight the complexity of the receptor involvement, especially TAAR1, in vascular regulation and emphasize the need for further mechanistic studies using more selective antagonists, receptor expression analyses, or knockout models. In general, our results demonstrate that TAAR1 agonists clearly have vasoactive effects as they may induce vasocontraction both directly and indirectly.

### Limitations and Future Perspectives

Interestingly, the AA and PEA analogues behaved differently in the three different vessel types with limited or inconsistent vasocontractile activity across the vessels. The four AA analogues did not show any effects in the mesenteric arteries and vasocontraction of the aorta and renal artery was induced inconsistently by most AA analogues. This contrasts with reports of dimethylamylamine which has been shown to produce vasopressor effects in vivo in rats through indirect sympathomimetic mechanisms, such as inhibition of noradrenaline reuptake [44, 45]. These discrepancies may reflect differences between in vitro isolated vessel assays and intact in vivo systems, where neural and hormonal feedback modulate vascular responses. This highlights one of the limitations of the current study.

Both male and female WKY rats were used in the present study. However, the study was not designed to detect sex-specific differences in vascular responses, as artery segments from multiple animals were distributed across experimental conditions. Future studies specifically powered to investigate sex differences in the vascular effects of phenethylamine analogues would therefore be valuable.

An important implication of our findings is that compounds commonly present in sports and weight-loss supplements can directly modulate vascular tone through adrenergic signaling pathways. Such effects may contribute to acute increases in vascular resistance and blood pressure, particularly in situations where sympathetic activity is already elevated, such as during exercise. By demonstrating direct vasocontraction in physiologically relevant vascular tissues, our findings provide an explanation between previously reported receptor activation and the cardiovascular adverse events described in case reports associated with these supplements.

Pharmacokinetic data of PEA and AA analogues in animals and humans remains scarce. For example, Haller et al. (2005) reported a mean plasma concentration of 2.8 ng/mL synephrine after ingestion of 47 mg synephrine, which is more than 50-fold lower than the EC_50_ determined for vasocontraction in the rat aorta in the present study (EC_50_ = 0.906 µM corresponding to 151 ng/mL) [46]. Our study is limited by the uncertainty regarding the reflection of the studied exposure concentration range to in vivo plasma concentrations, since there is lack of robust pharmacokinetic data. Maximal daily doses of 968 mg PEA, 980 mg BMPEA, 480 mg DMPEA, 226 mg *p*-synephrine and 130 mg *p*-octopamine have been reported in supplements (Table 2), but data on plasma concentrations after ingestion of these doses are lacking [3, 4, 47]. In contrast, maximal concentrations of 0.302 µM were detected in human plasma after oral intake of 25 mg of the BMPEA-isomer amphetamine [48]. Given the structural similarity, BMPEA and its analogues may share pharmacokinetic properties and similar plasma concentrations, which suggests that the effective concentration of BMPEA in the current study (EC_50_ = 16.9 µM) could fall within a physiologically relevant range of plasma concentrations, thereby resulting in in vivo effects.

**Table 2:** Detected amounts of PEA (analogues) (mg/day) in food supplements. N.D. indicates no data is available on the quantification

Food supplement ingredient	Dose (mg/day)	Reference
*p*-synephrine	0.00903–226	[3, 4, 49–52]
*p*-octopamine	0.07–130	[3, 4, 49, 51, 52]
halostachine	N.D	
BMPEA	91.7–980	[4, 5, 53, 54]
PEA	127–968	[3, 4]
DMPEA	6.8–480	[4, 54]

Additionally, the lack of pharmacokinetic data complicates conducting a reliable risk assessment of PEA and AA analogues exposure in humans. This is particularly concerning given the widespread popularity and use combined with the lack of harmonized safety regulations [1–3]. These gaps highlight the urgent need for human pharmacokinetic and biomonitoring studies to protect consumer health. An additional challenge is the complex composition of pre-workout and weight loss supplements, frequently containing pharmacologically active ingredients [3]. This complicates both (clinical) research and regulatory assessment, emphasizing the necessity for an effective prioritization strategy. In previous work, we used a physiologically based kinetic model to extrapolate in vitro potencies for ADR and TAAR1 activation to estimated human effective doses [55]. The current study extends these findings by demonstrating broader physiological effects, namely vasocontraction, beyond receptor activation. Notably, the potency values obtained in the current study are in the same range as those reported for human ADR and TAAR1 activation [9]. This suggests that our vascular rat model provides a physiologically relevant translation to human in vitro data. A further limitation is that only single compounds were tested, whereas real-life exposure involves mixtures. Further research should also address these co-exposures, since pre-workout supplements often contain cocktails of PEA and AA analogues combined with high doses of caffeine (up to daily doses of 1940 mg), raising the possibility for additive and synergistic effects on the cardiovascular system [3, 56]. Such interactions may lower the threshold at which adverse events occur, particularly in sensitive populations such as individuals with pre-existing cardiovascular disease or those carrying out strenuous exercise. It is plausible that the vascular effects observed in the present study may therefore be enhanced under pathological conditions such as hypertension or endothelial dysfunction. As such, it would be valuable for future research to study the effects of the PEA and AA analogues as well in diseased models, such as isolated arteries of spontaneously hypertensive rats and isolated blood vessels with endothelial dysfunction by inhibiting nitric oxide synthase or removal of the endothelium. In addition, exercise could be mimicked by exposure to enhanced catecholamine levels or electrical field stimulations. Moreover, the current study extensively investigated the impact of the food supplement ingredients on arterial vascular beds due to their central role in regulating systemic vascular resistance and blood pressure. However, venous tone also plays an important role in cardiovascular regulation by modulating venous return and cardiac preload and output [57]. Future studies investigating the effects of PEA analogues in venous tissue could therefore provide additional insight into their systemic hemodynamic effects.

### Conclusion

In this study we demonstrate that multiple PEA and AA analogues can directly induce vasocontraction of functionally distinct vascular beds (mesenteric artery, renal artery and aorta segments) via various mechanisms. These compounds are ingredients of very popular pre-workout supplements and mainly used by (amateur) athletes and/or overweight individuals to enhance their workout performance. The demonstrated effects in this study imply potentially deleterious consequences for cardiovascular health in humans. These risks may be particularly pronounced in exercising individuals that have an already activated sympathetic nervous system or individuals with underlying cardiovascular pathologies. Our study highlights the need for more research on the pharmacodynamics and pharmacokinetics of these compounds in humans in order to establish a thorough risk assessment of pre-workout supplements containing PEA and AA analogues.

## Data Availability

Data will be available on request.

## Appendix

See Figs. 5, 6 and 7.
